# Age- and Phenotype-Dependent Changes in Circulating MMP-2 and MMP-9 Activities in Normotensive and Hypertensive Rats

**DOI:** 10.3390/ijms21197286

**Published:** 2020-10-02

**Authors:** Marta Kollarova, Angelika Puzserova, Peter Balis, Dominika Radosinska, Lubomira Tothova, Monika Bartekova, Miroslav Barancik, Jana Radosinska

**Affiliations:** 1 Institute of Physiology, Faculty of Medicine, Comenius University in Bratislava, 811 08 Bratislava, Slovakia; marta.husseinova@fmed.uniba.sk (M.K.); monika.bartekova@savba.sk (M.B.); 2 Centre of Experimental Medicine, Slovak Academy of Sciences, Institute of Normal and Pathological Physiology, 813 71 Bratislava, Slovakia; angelika.puzserova@savba.sk (A.P.); peter.balis@savba.sk (P.B.); 3 Faculty of Natural Sciences, Comenius University in Bratislava, 842 15 Bratislava, Slovakia; dominikaradosinska@gmail.com; 4 Institute of Molecular Biomedicine, Faculty of Medicine, Comenius University in Bratislava, 811 08 Bratislava, Slovakia; tothova.lubomira@gmail.com; 5 Centre of Experimental Medicine, Slovak Academy of Sciences, Institute for Heart Research, 841 04 Bratislava, Slovakia; miroslav.barancik@savba.sk

**Keywords:** matrix metalloproteinases, gelatinases, hypertension, aging, spontaneously hypertensive rats

## Abstract

Matrix metalloproteinases (MMPs) are important in the pathogenesis of numerous diseases. The present study aimed to monitor the activation of MMP-2 and MMP-9 in spontaneously hypertensive rats (SHR) and their normotensive counterparts—Wistar-Kyoto rats (WKY). The animals were divided according to age (7, 20, and 52 weeks) and phenotype into: WKY-7, WKY-20, WKY-52, SHR-7, SHR-20 and SHR-52 groups. MMP plasma activities were determined by gelatine zymography. We monitored selected parameters of oxidative stress and antioxidant status. N-terminal pro-brain natriuretic peptide (NT-proBNP) was determined as a marker of heart function and neurohumoral activation. SHR-7 showed higher MMP-2 activity compared with WKY-7, while SHR-52 showed lower MMP-2 and MMP-9 activities compared with WKY-52. Examining age-dependent changes in MMP activities, we found a decrease in MMP-2 activity and increase in MMP-9 activity with increasing age in both phenotypes. Parameters of oxidative stress and antioxidant status as well as NT-proBNP levels were not significantly worsened due to aging in SHR. Our results suggest that hypertension is accompanied by varying MMP activation during aging. The results of our study may indicate that MMP-2 inhibition is therapeutically applicable during the development of hypertension, while in developed, stabilized and uncomplicated hypertension, systemic MMP-2 and MMP-9 inhibition may not be desirable.

## 1. Introduction

Hypertension is a major risk factor for renal, cardiovascular, and cerebrovascular disease, and a leading underlying cause of global mortality and morbidity. The main goal of treating hypertension is to reduce morbidity and mortality due to this disease. Treatment is twofold—pharmacological and non-pharmacological—which are mostly combined [1,2]. Hypertension has a complex pathophysiological background whereby genetic and environmental factors combine with a plethora of physiological pathways and mechanisms to ultimately yield the phenotype. Epidemiological studies have improved the understanding of environmental factors (e.g., diet and exercise), but it is difficult to identify the exact role of genetics in cases with the same environment in families [3]. Essential hypertension affects up to 95% of patients with high blood pressure (BP) and has no apparent cause. In the study of Pazoki et al. (2018), it was shown that a healthy lifestyle is associated with lower BP levels and a lower risk of subsequent cardiovascular events within each category of BP genetic profile. A high genetic risk was largely offset by a favorable lifestyle, but the people with low genetic risk could lose their inherent protection due to an unhealthy lifestyle. These findings highlight the need for timely lifestyle interventions to offset the lifetime risk of future high BP and cardiovascular diseases (CVD) [4]. Matrix metalloproteinases (MMPs) are proteolytic enzymes important for degradation and remodelling the extracellular matrix (ECM) under both physiological and pathological conditions. MMPs can influence disease development through three different pathophysiological mechanisms: tissue destruction, fibrosis, and ECM degradation. Many studies showed that MMPs are directly associated with almost every biological process that involves ECM remodeling, from embryogenesis to cell death, and also could be regulators of intercellular signaling in various tissues [5,6,7]. In humans, more than 20 MMPs are known, divided into six groups according to their structure and substrate specificity [8,9]. MMP-2 and MMP-9 belong to a group of gelatinases and are involved in the pathogenesis of a wide range of CVD [10].

Although MMP-2 and MMP-9 belong to one group, there are some differences. MMP-2 is produced by fibroblasts, endothelial cells, and osteoblasts and is involved in physiological remodelling of tissue (e.g., during growth). MMP-9 is released from polymorphonuclear leukocytes, epithelial cells and activated monocytes resp. macrophages that are escalated with pathological stimuli [7,11]. Both MMP-2 and MMP-9 are able to cleave type I, V, VII, and XI collagen and laminin. Due to this ability, they participate in the remodelling of the basement membrane and their activity regulates cell migration and proliferation during wound healing and tumour growth [12,13]. While the MMP-9 gene has proinflammatory transcription factor binding sites, these binding sites are not present in the case of gene for MMP-2. MMP-2 is constitutively expressed by a full spectrum of cells without necessary stimulation. Even MMP-9 is constitutively expressed in eosinophils and neutrophils; the main producers are macrophages after their activation by the stimulus. Thus, MMP-9 is more related to the immune system, as most of MMP-9 is released from activated immune cells [6]. MMP-9 has a similar role to MMP-2; both are involved in the proteolytic activation of many cytokines [13,14]. The role of MMP-2 and MMP-9 in the etiopathogenesis of essential hypertension is documented in a number of studies, but none of them comprehensively address their age-dependent activation [15,16,17].

In the current study, we aimed to describe changes in plasma MMP-2 and MMP-9 activities in the most-used experimental model of essential hypertension—spontaneously hypertensive rats (SHR), depending on the age of the experimental animals (7-, 20- and 52-week-old ones: SHR-7, SHR-20, and SHR-52) and their normative control of Wistar-Kyoto rats (WKY: WKY-7, WKY-20, and WKY-52). Since the direct activation of MMPs occurs under conditions of increased oxidative stress [14], the activities of circulating MMP-2 and MMP-9 were monitored along with parameters reflecting the oxidative stress and antioxidant capacity.

## 2. Results

### 2.1. Basic Characteristics of Experimental Animals—Body Weight, Systolic Blood Pressure and Heart Rate

**Figure 1:**
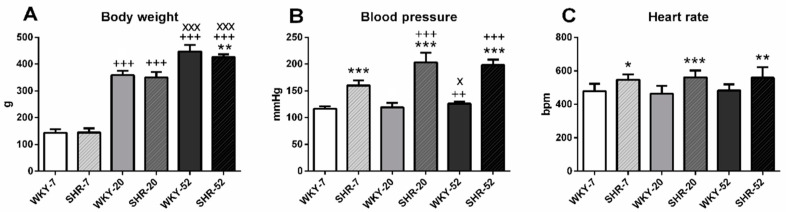
Basic characteristics of experimental animals: body weight (**A**), systolic blood pressure (**B**), and heart rate (**C**). The data are presented as means ± SD. Statistical significance: * *p* < 0.05, ** *p* < 0.01, *** *p* < 0.001 between WKY and SHR at the same age (WKY-7 vs. SHR-7, WKY-20 vs. SHR-20 and WKY-52 vs. SHR-52), ++ *p* < 0.01, +++ *p* < 0.001 vs. 7-week group of the same phenotype (WKY-20 vs. WKY-7, SHR-20 vs. SHR-7, WKY-52 vs. WKY-7 and SHR-52 vs. SHR-7), ^X^ *p* < 0.05, ^XXX^ *p* < 0.001 vs. 20-week group of the same phenotype (WKY-52 vs. WKY-20 and SHR-52 vs. SHR-20). WKY—Wistar-Kyoto rats, SHR—Spontaneously hypertensive rats, bpm—beats per minute.

Body weight (BW) was lower in SHR-52 compared with age-matched WKY, while it was not different between the WKY and SHR in 7- and 20-week-old animals. Considering the age of WKY and SHR, BW was significantly lower in 7-week-old animals than in 20-week-old ones and it was also lower in 20-week-old animals than in 52-week-old ones (Figure 1A). Systolic blood pressure (BP) was significantly higher in all SHRs compared with the age-matched WKY animals. Comparing the WKY groups separately, BP was significantly lower in WKY-7 than in WKY-52 and it was also lower in WKY-20 than in WKY-52. Comparing the SHR groups only, BP in SHR-7 was lower than in SHR-20 and SHR-52 (Figure 1B). Heart rate (HR) was higher in all SHRs compared with age-matched WKY rats (Figure 1C).

### 2.2. Markers of Oxidative Stress

#### 2.2.1. Markers of Oxidative Damage of Proteins and Lipids

**Figure 2:**
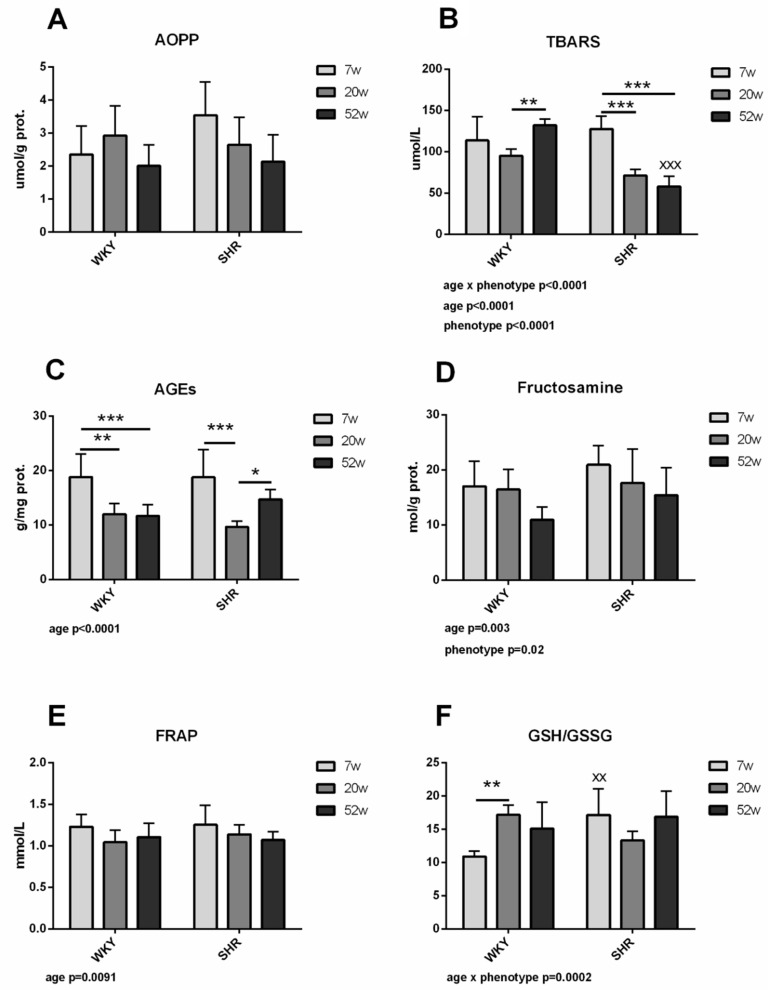
Markers of oxidative stress: markers of oxidative damage—AOPP (**A**) and TBARS (**B**), carbonyl stress—AGEs (**C**) and fructosamine (**D**) and antioxidant capacity—FRAP (**E**) and GSH/GSSG (**F**). The data are presented as means ± SD. Statistical significance: * represents *p* < 0.05, ** represents *p* < 0.01 and *** *p* < 0.001. ^XX^ represents *p* < 0.01 and ^XXX^ *p* < 0.001 between the age-matched different phenotypes. WKY—Wistar-Kyoto rats, SHR—spontaneously hypertensive rats, AOPP—advanced oxidation protein products, TBARS—thiobarbituric acid reactive substances, AGEs—advanced glycation end-products, FRAP—ferric-reducing antioxidant power, GSH—reduced glutathione, GSSG—oxidized glutathione.

Two-way analysis of variance (ANOVA) did not reveal significant differences between experimental groups in advanced oxidation protein products (AOPP) concentrations for both factors (age/phenotype). In addition, there were no significant differences in AOPP levels between groups (Figure 2A). Both factors (age and phenotype) significantly impacted thiobarbituric acid reactive substances (TBARS)—they were higher in WKY than in SHR, and concentrations decreased with age in SHR. Interaction age x phenotype was also significant. TBARS were significantly higher in WKY-52 compared with SHR-52. Comparing the WKY groups separately, TBARS were significantly lower in WKY-20 than in WKY-52. Comparing the SHR groups only, TBARS were higher in SHR-7 than in SHR-20 and SHR-52 (Figure 2B).

#### 2.2.2. Carbonyl Stress

Advanced glycation end-products’ (AGEs) concentrations initially decreased with age. AGEs were significantly higher in WKY-7 than WKY-20 and WKY-52, while there was no difference between WKY-20 and WKY-52. In SHR, AGEs were significantly higher in SHR-7 than in SHR-20 but significantly lower in SHR-20 than in SHR-52. A 52-week SHR had similar concentrations of AGEs when compared to SHR-7 (Figure 2C), suggesting an increase in AGEs with further aging in hypertensive animals. Fructosamine concentrations were without significant differences between individual groups (Figure 2D).

#### 2.2.3. Antioxidant Capacity

There were no significant differences between individual groups when evaluating ferric-reducing antioxidant power (FRAP) as antioxidant marker (Figure 2E). Regarding the ratio of reduced to oxidized glutathione (GSH/GSSG), the interaction between age and phenotype was significant. GSH/GSSG was lower in WKY-7 comparing with SHR-7. Comparing the WKY groups separately, WKY-7 had lower GSH/GSSG ratio than WKY-20 (Figure 2F).

### 2.3. N-Terminal Pro-Brain Natriuretic Peptide Concentration

**Figure 3:**
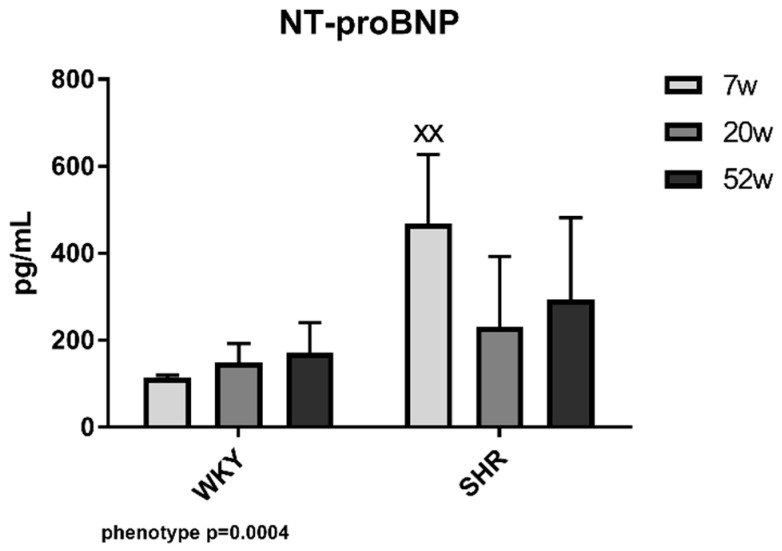
NT-proBNP in WKY and SHR in different age groups. The data are presented as means ± SD. Statistical significance: ^XX^ represents *p* < 0.01 in comparison with age-matched WKY. WKY—Wistar-Kyoto rats, SHR—spontaneously hypertensive rats NT-proBNP—N-terminal pro-brain natriuretic peptide.

In N-Terminal Pro-Brain Natriuretic peptide (NT-proBNP), concentrations in SHR-7 were significantly higher compared with WKY-7 (Figure 3).

### 2.4. Circulating MMPs Activities

#### 2.4.1. Phenotype-Dependent Analyses

**Figure 4:**
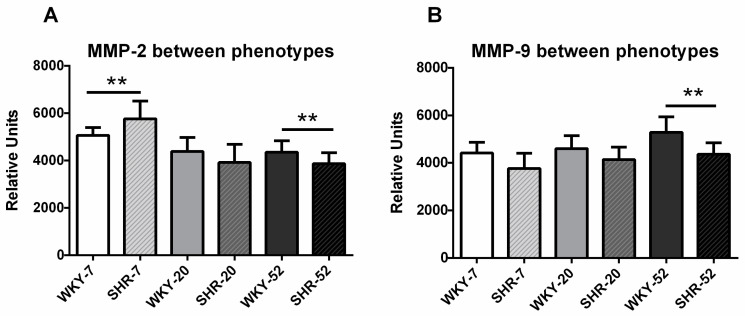
Phenotype differences in MMP-2 (**A**) and MMP-9 (**B**) activity. The data are presented as means ± SD. Statistical significance: ** represents *p* < 0.01. MMP—matrix metalloproteinase, WKY—Wistar-Kyoto rats, SHR—spontaneously hypertensive rats.

MMP-2 activity was different between phenotypes in two age categories—it was lower in WKY-7 than in SHR-7, and it was higher in WKY-52 than in SHR-52. In 20-week-old rats, there were no differences in plasmatic MMP-2 activities between normotensive and hypertensive animals (Figure 4A).

MMP-9 activity was higher in WKY-52 than in SHR-52. In 7-week-old rats as well as in 20-week-old rats, there were no differences in plasmatic MMP-9 activities between normotensive and hypertensive animals (Figure 4B).

#### 2.4.2. Age-Dependent Analyses

**Figure 5:**
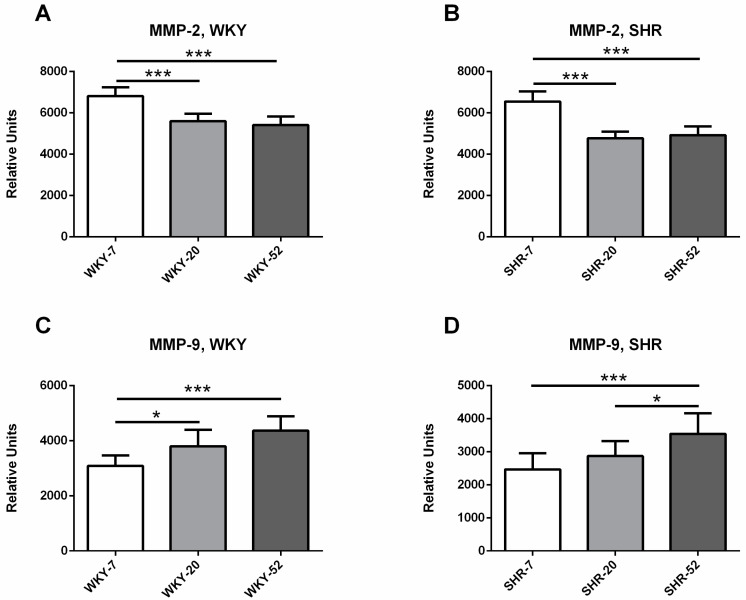
Age differences in the activity of MMP-2 in WKY (**A**) and SHR (**B**) and age differences in the activity of MMP-9 in WKY (**C**) and SHR (**D**). The data are presented as means ± SD. Statistical significance: * represents *p* < 0.05; *** represents *p* < 0.001. MMP—matrix metalloproteinase, WKY—Wistar-Kyoto rats, SHR—spontaneously hypertensive rats.

MMP-2 activity was significantly higher in WKY-7 compared with WKY-20 and in WKY-7 compared with WKY-52 (r = −0.73, *p* < 0.0001, *n* = 43). The analogical differences were observable in SHR—MMP-2 activity was significantly higher in SHR-7 compared with SHR-20 and in SHR-7 compared with SHR-52 (r = −0.66, *p* < 0.0001, *n* = 42) (Figure 5A,B).

MMP-9 activity was significantly lower in WKY-7 compared with WKY-20 and in WKY-7 compared with WKY-52 (r = 0.75, *p* < 0.0001, *n* = 43). In SHR, MMP-9 activity was significantly lower in SHR-7 compared with SHR-52 and in SHR-20 compared with SHR-52 (r = 0.64, *p* < 0.001, *n* = 42) (Figure 5C,D).

## 3. Discussion

The role of circulating MMP-2 and MMP-9 in the etiopathogenesis of essential hypertension has not yet been studied in connection with age [15,16,17]. We monitored MMP-2 and MMP-9 activities along with oxidative stress and antioxidant capacity parameters due to possible direct MMP activation under conditions of increased oxidative stress [18]. According to our knowledge, studies exploring the reference values of oxidative stress markers in the plasma and activities of MMP-2 and MMP-9 in plasma in three age categories of SHR and WKY rats have not been published to date.

A key role among the potential factors involved in the pathogenesis of hypertension may be ascribed to the cardiac natriuretic peptide (NP) system. Previous genomic association studies have found that genes for BNP and natriuretic peptide receptor C are among the most important genes involved in the pathogenesis of essential hypertension, and none of the renin–angiotensin–aldosterone system genes have been identified as contributing to the polygenicity of essential hypertension [19]. Investigation of MMPs in experimental models as well as in hypertensive patients is of clinical interest also in terms of their use as potential biomarkers of disease progression and potential predictors of treatment efficacy. Research into ECM-dependent pathophysiology can lead to advances in the diagnosis and treatment of subsequent CVD [16,20,21,22].

### 3.1. Biometric and Cardiovascular Parameters

Our animal model of essential hypertension exhibited features typical for SHR and their BP, HR, and BW when compared with WKY. Although BW and BP increased with age in both groups, HR was stable. Interestingly, we observed an age-related BP increase in 52-week-old normotensive WKY rats. In 7-week-old SHR, hypertension was not fully developed yet; it remained stable from 20 weeks of age. It is known that BP starts to increase spontaneously from around 5 to 6 weeks of rat life in the SHR strain. It raises by the age of 12 weeks, and, afterwards, arterial BP stabilizes [23].

### 3.2. Oxidative Stress Parameters

Oxidative stress is found to be associated with both hypertension and aging [24,25,26]. In our experiment, we monitored selected parameters of oxidative stress and antioxidant status in order to comprehensively understand age- and phenotype-dependent changes in our experimental animals. TBARS, as a marker of lipid peroxidation [27], lowered with age in SHR when compared to WKY. Likewise, the products of carbonyl stress—AGEs [28]—were lower in 20-week and 52-week WKY and SHR animals when compared to corresponding young animals. We cannot fully explain the reason why these two markers, generally believed to be increased with aging [29,30], were found to be decreased. Nonetheless, it is noteworthy that the GSH/GSSG ratio was higher in 7-week SHR rats when compared to 7-week WKY rats, and this increase persisted in 20-week and 52-week animals. It is thus our hypothesis that young SHR animals have compensatory mechanisms with increased antioxidative status as a result, which in turn leads to decreased oxidative stress, at least for some period. Even though a 52-week rats can be considered as aged, this still represents half a rat’s lifespan, and the antioxidant reserves does not have to be exhausted yet. On the other hand, the measured markers of oxidative stress and antioxidant status represent only few substances that were measured. Moreover, AOPP, fructosamine and FRAP did not show any dynamics or differences within or between the groups. The determination and evaluation of oxidative stress includes a broad number of markers to be measured, and as such represents a real challenge [31]. Therefore, it is also possible that, while our results suggest decreased oxidative stress in aged SHR animals, several other not-measured parameters would point to increased oxidative stress. Unfortunately, there are no other relevant studies to compare our results with, either because of inappropriate age [32,33] or the used model [34].

To date, no studies have been known that focus on WKY and SHR in different age groups and these parameters.

### 3.3. NT-proBNP

The main stimulus for cardiac NT-proBNP production is stretch of cardiomyocytes. In addition, it could offer information about the neurohumoral activation of experimental animals [35]. It was expected and confirmed in our experiment that NT-proBNP levels are higher in SHR when compared with WKY rats. Surprisingly, NT-proBNP concentration seems not to be changed due to aging in our hypertensive animals. This might be explained as a neurohumoral adaptation to high BP after stabilisation of its increase in this model of essential hypertension. It is worth adding that our 52-week-old experimental animals did not show any symptoms of heart failure. We could predict that, with further aging and cardiac function deterioration, NT-proBNP will consequently rise, as was observable in a previous study [36].

### 3.4. MMPs

In vitro studies have shown that MMP-2 may play an important role in the regulation of BP by the cleavage of bigendothelin-1 to a potent endothelin-1 vasoconstrictor factor. MMP-2 in vitro could also cleave a vasodilator calcitonin gene-related peptide that produces degradation products with lower vasodilator activity, and could also act on adrenomedullin, which acts as a vasoconstrictor [37]. Elevated levels and activities of MMP-2 have been observed in vascular remodelling due to hypertension. Phenotypic changes in vascular smooth muscle cells (VSMCs) include their proliferation, migration and subsequent vascular wall remodelling. In the animal study, Belo et al. (2016) show that increased arterial activity of MMP-2 directly contributes to the reduction in calponin-1 levels and induces VSMC proliferation before the onset of hypertension-induced chronic arterial remodelling [38]. The detection of elevated MMP-2 levels in hypertensive patients could potentially serve as an early marker of vascular remodelling. For MMP-9, its high serum levels are likely responsible for the cleavage of β2-adrenergic receptors on endothelial cells and indicate a faster progression of hypertension in humans [20]. It may be presumed that because MMP-2 is more involved in physiological remodelling, e.g., during tissue growth, its activity is less needed by age. In contrast, MMP-9 is more activated in aging because of pathological remodelling.

Our experimental model showed differences in the activities of MMP-2 between SHR and WKY rats in 7- and 52-week old rats. In 7-week-old animals, i.e., during the development of hypertension, we were able to observe higher MMP-2 activation in SHR when compared with WKY rats. Such a difference was not observable in plasma MMP-9 activity. This could point to a more important role of MMP-2 in the development of hypertension in comparison with MMP-9 in this particular model. After stabilization of hypertension, i.e., in 20-week-old animals, plasma MMP-2 activity is the same in WKY rats and in SHR. With further aging, MMP-2 becomes lower in SHR-52 than in WKY-52. There is a parallel with a study describing humans, in which stabilized arterial hypertension was accompanied by the decrease in plasma MMP-2 activity in comparison with normotensive controls [39]. Similarly, in 52-week-old hypertensive rats, the plasma MMP-9 activity was lower than in age-matched control WKY rats. Many drugs that are administered to hypertensive patients also affect MMPs, namely MMP-2 and MMP-9 [40]. We can see that the activities of MMPs depend on age and may interfere with treatment.

### 3.5. Conclusion

In both phenotypes, the activity of MMP-2 decreased with age. In contrast, MMP-9 activity increased with age. The results of our study may indicate that MMP-2 inhibition is therapeutically applicable during development of hypertension, while in developed, stabilized and uncomplicated hypertension, systemic MMP-2 and MMP-9 inhibition may not be desirable.

## 4. Materials and Methods

### 4.1. Animals

We used males of SHR (sublines SHR/NHsd, HARLAN UK) and WKY (sublines WKY/NHsd, HARLAN UK) rats. All rats were born in the certified animal facility (Centre of Experimental Medicine, Institute of Normal and Pathological Physiology, Slovak Academy of Sciences) to maintain the same environmental background for all the animals. The rats were housed at constant room temperature (22–24 °C) and humidity (45–60%) with a 12 h light–dark cycle (lights on from 06:00 a.m. to 06:00 p.m.) and fed a standard pellet diet with tap water ad libitum. Rats were divided into 6 groups according to age (7, 20 and 52 weeks) and strain into SHR-7 (*n* = 7), SHR-20 (*n* = 7), SHR-52 (*n* = 10) and WKY-7 (*n* = 10), WKY-20 (*n* = 7), WKY-52 (*n* = 8). The following parameters were monitored throughout their life: BW, HR, and systolic BP. When the rats reached the determined age, they were exposed to brief CO_2_ anaesthesia and subsequently killed by decapitation. Trunk blood was collected in heparinized test tubes. Blood was immediately centrifuged (850× *g*, 10 min, 4 °C) and blood plasma was stored at −80 °C till the analysis.

All procedures were performed in accordance with the institutional and European Guidelines on Laboratory Animal Care and approved by the Ethics Committee of the Centre of Experimental Medicine Slovak Academy of Sciences (project code: EK/1/17, approved 6 February 2017 and EK/vekhyp/2014, approved 24 June 2014) and by the Department of Animal Wellness, State Veterinary and Food Administration of the Slovak Republic (decision No. Ro-1087/17-221 and No. Ro-3095/14-221).

### 4.2. Measurements of Systolic Blood Pressure and Heart Rate

Systolic BP and HR were measured in preconditioned, conscious rats by non-invasive tail-cuff plethysmography (using the Statham Pressure Transducer P23XL, Hugo Sachs, March-Hugstetten, Germany) between 08:00 a.m. and 11:00 a.m. Before the measurement, the rats were handled and accustomed to the tail-cuff procedure for BP recording during three separate sessions. Each session included handling, heating of rats for approximately 4 min at 33–34 °C in a warm chamber and placement of the rat into a restrainer of appropriate size for the determination of BP. Each value was calculated as the average value of five to six successful measurements, excluding the first measurement. BW was recorded at the same time.

### 4.3. Biochemical Analysis of Oxidative Stress and Antioxidant Status

Markers of oxidative damage products, antioxidant capacity, and the presence of a general marker of oxidative stress were determined in plasma samples. Measurements were performed on a TecanSafire II Instrument (Grödig, Austria). Chemicals used in the analysis were purchased from Sigma-Aldrich (Steinheim, Germany). We proceeded according to previous studies [41,42].

We measured markers of oxidation damage: markers of protein oxidation—AOPP, markers of lipid peroxidation—TBARS, marker of carbonyl stress—AGEs, and fructosamine. FRAP was measured in plasma as the marker of antioxidant status. GSH/GSSG ratio was measured as the general marker of oxidative stress.

Briefly: AOPP: 200 µL of samples and standards (chloramine T mixed with potassium iodide) was mixed with 20 µL of glacial acetic acid. Absorbance was measured at λ = 340 nm. TBARS: 20 µL of samples and standards (1,1,3,3-tetraethoxypropane) was mixed with 30 µL of distilled water, 20 µL of 0.67% thiobarbituric acid and 20 µL of glacial acetic acid. The microplates were vortexed and incubated (45 min, 95 °C). A total of 100 µL of n-butanol was added into the samples and microplates were centrifuged (10 min, 4 °C, 2000× *g*). A total of 70 µL of the upper phase was transferred into a new plate. Fluorescence was measured at λ_ex_ = 515 nm and λ_em_ = 535 nm. AGEs: 20 µL of samples and standards (AGE-BSA) was mixed with 180 µL of phosphate-buffered saline in the dark microtiter plate. Fluorescence was measured at λ_ex_ = 370 nm and λ_em_ = 440 nm. Fructosamine: 20 µL of samples and standards (16 mmol/L 1-deoxy-morpholino-D-fructose) was mixed with 100 µL of 0.25 mmol/L nitro blue tetrazolium containing 0.1 mmol/L sodium carbonate buffer (pH 10.35) and 1 mmol/L nitroblue tetrazolium. Microplates were incubated (37 °C, 15 min) and absorbance was measured at λ = 530 nm. FRAP: 200 μL of FRAP reagent (acetate buffer, pH 3.6, tripyridyl-s-triazine, FeCl_3_·6H_2_O, distilled water, all warmed at 37 °C) was pipetted into a microplate. Absorbance was measured as blank at λ = 530 nm. Afterwards, 20 µL of samples and standards (100 mmol/L FeSO_4_·7H_2_O) was added and absorbance was measured at λ = 530 nm. GSH: 10 µL of samples was mixed with 10 µL of the ophthaldehyde solution (1 mg/mL) and 180 µL of the phosphate buffer solution (100 mmol/L with 2.5 mM EDTA-Na2). Microplates were incubated (room temperature, 15 min) and fluorescence was measured at λ_ex_ = 350 nm and λ_em_ = 460 nm. GSSG: 25 µL of the samples was mixed with 10 µL of N-ethylmaleimide (5 mg/mL) and incubated (room temperature, 40 min). A total of 10 µL of the mixture was transferred into the new microplate. A total of 10 µL of the ophthaldehyde and 180 µL of NaOH 0.1 mmol/L was added and microplates were incubated (room temperature, 15 min). Fluorescence was measured at λ_ex_ = 350 nm, λ_em_ = 460 nm.

The protein concentration was measured by the bicinchoninic acid kit (Sigma-Aldrich, Munich, Germany), according to the manufacturer’s instructions. The bovine serum albumin was used as standard.

### 4.4. Analysis of NT-proBNP

Levels of NT-proBNP were measured by the Rat NT-proBNP ELISA Kit (Elabscience Biotechnology Inc., Houston, TX, USA), according to the manufacturer’s instructions.

### 4.5. Analysis of MMPs Activity

The activities of circulating MMP-2 and MMP-9 were evaluated by using a gelatine zymography in 10% polyacrylamide gel containing gelatine (2 mg/mL) as a substrate [43]. The samples were suspended in Laemmli buffer (without 2-mercaptoethanol) and pipetted into a gel. Zymography involves the electrophoretic separation of proteins under denaturing but not reducing conditions in a polyacrylamide gel. After electrophoresis, the gel was washed twice for 20 min with 50 mmol/L Tris-HCl (pH 7.4), containing 2.5% Triton X-100, and then incubated overnight at 37 °C in a substrate buffer to examine specific proteinases containing 50 mmol/L Tris-HCl (pH 7.4), 10 mmol/L CaCl_2_ and 1.25% Triton X-100. After incubation, the gel was stained with 1% Coomassie Brilliant Blue G-250 and destained with 40% methanol and 10% acetic acid solution. The gelatinolytic activities of the MMP-2 and MMP-9 were detected as clear bands on a blue background. The intensity of these bands was assessed by using ImageJ program (NIH, Bethesda, MD, USA). Due to the method used, we were limited in the count of samples processed in one gel/zymogram. Thus, phenotypic differences and age differences were separately and independently evaluated.

### 4.6. Statistical Analyses

The normality of the data was analysed by the D’Agostino-Pearson omnibus test. Outliers were detected using the Grubbs’ test. The data are presented as the mean ± standard deviation (SD). Statistical significance for BW, BP, HR, oxidative status, and NT-proBNP was analysed by 2-way ANOVA for factors age and phenotype (i.e., hypertension). Due to the methodology for determining MMPs activity, from which we obtained semi-quantitative data, we separately evaluated the phenotype (1-way ANOVA and Tukey’s multiple comparisons post-hoc test) and separately the effect of age (*t*-test). Pearson correlation coefficients were calculated to explore the relationships between variables. Values were considered to differ significantly when *p* < 0.05. For all statistical analyses, GraphPad Prism 7.02 (GraphPad Software, Inc., San Diego, CA, USA) was used.

## 5. Conclusions

Hypertension is accompanied by varying activation of gelatinases at young and old age. If the same tendency in humans is confirmed, it could be used in a different therapeutic approach to hypertensive patients of different ages.

## Abbreviations

AGEs	Advanced glycation end-products
ANOVA	Analysis of variance
AOPP	Advanced oxidation protein products
BP	Blood pressure
bpm	Beats per minute
BW	Body weight
CVD	Cardiovascular diseases
ECM	Extracellular matrix
FRAP	Ferric reducing antioxidant power
GSH	Reduced glutathione
GSSG	Oxidized glutathione
HR	Heart rate
MMPs	Matrix metalloproteinases
NP	Natriuretic peptide
NT-proBNP	N-terminal pro-brain natriuretic peptide
SD	Standard deviation
SHR	Spontaneously hypertensive rats
TBARS	Thiobarbituric acid reactive substances
VSMCs	Vascular smooth muscle cells
WKY	Wistar-Kyoto rats
